# Supplementary material for: Prevalence and risk factors for Trichomonas vaginalis infection among adults in the U.S., 2013–2014
Source: PLoS One. 2020 Jun 16;15(6):e0234704. doi: 10.1371/journal.pone.0234704 (PMC7297358; doi:10.1371/journal.pone.0234704)
Supplement: Attachment — Submitted filename: Response to Reviewers.docx [file pone.0234704.s001.docx]

Reviewer #1: Overall, nice report that will contribute to then literature on TV. Just a few very minor suggestions below, the primary concern at the moment is the introduction need (probably) just one more paragraph.

1. Review the intro for typos, there are just a few in there. Thank you -- corrections have been made to most recent copy of the manuscript.
2. Even for me, the introduction may be a bit brief. Are there other studies that have examined demographic and behavioral characteristics? Could you summarize these some here? How is your study adding to past literature? I appreciate your comments -- I added additional background information and three resources (two of them newly published since this paper’s submission) which I feel added to the paper overall.
3. Table 1, please provide total N in title. This was added (n = 4057)
4. Table 3, footnote a seems unnecessary, but up to the authors.

We left the footnote below the table to indicate to readers that the listed characteristics (all variables in the table) were included in the adjusted odds ratio (none were left out).
5. Table 3, footnote b, is there a concentration threshold that should be specified here?

It is important to specify serum cut points for cotinine concentration because they vary slightly by gender and ethnicity. These cut points are listed in the results section, lines 136-140.
6. Results, paragraph 2, I would cite the 2001-04 NHANES cycle here. Thank you, this was added.

Reviewer #2: In the manuscript “Prevalence and risk factors for asymptomatic Trichomonas vaginalis infection among adults in the U.S., 2013-2014” (PONE-D-19-34688), Tompkins et al. analyze a nationwide (asymptomatic) T. vaginalis prevalence assessment that was conducted under the auspices of the 2013-2014 National Health and Nutrition Examination Survey (NHANES). Noteworthy within this survey is the fact that nucleic acid amplification testing (in particular, transcription-mediated amplification) was used for laboratory diagnosis of T. vaginalis infection, rather than wet mount microscopy (as was used in past assessments). In this report, demographic associations with T. vaginalis detection included female gender, non-Hispanic blacks, individuals below the federal poverty level, and active smokers. No associations were made with participant age, as have been made in other published studies. Overall infection prevalence was reported at 1.2%, which the authors (in the Discussion section) extrapolated to 2.1 million individuals.

The manuscript was well written. Statistical methods appear appropriate. This report may potentially contribute valuable information toward national prevalence estimates of trichomoniasis. Specific comments follow:

1) The authors report an assessment of asymptomatic trichomoniasis. How did the authors determine that the patients were truly asymptomatic? Such documentation should be presented within the manuscript; After reviewing the 2013-2014 CDC (NHANES) questionnaire again, it probably does not ask specific enough questions to ascertain whether or not participants are truly asymptomatic from an STI, as it does not ask questions specifically regarding dysuria, for example. Participants report existing conditions, but as it does not specifically ask about existing STIs, we omitted “asymptomatic” from the title of the manuscript.
2) On which automated (Hologic) system were the APTIMA Trichomonas vaginalis Assays performed? As the authors likely know, male urine testing is FDA-cleared on TIGRIS instrumentation, while Panther automation (to the knowledge of this Reviewer) does not possess this indication. If performed with Panther automation, the authors may need to provide verification data, as such testing would be considered a laboratory-modified test. If performed on the TIGRIS, then the authors need to state as such.

The laboratory manual from CDC (<https://wwwn.cdc.gov/nchs/data/nhanes/2013-2014/labmethods/TRICH_H_MET_TRICHOMONAS.pdf>) states that the test was performed on the Panther system. The authors did not collect the data, and after review of the manual, it appears that the CDC performed quality control checks within their lab protocol. The NHANES dataset was downloaded from CDC and is open source, and was used for analysis in this manuscript. The lab analysis was performed by the Division of AIDS, STD, and TB Laboratory Research National Centers for Infectious Diseases National Centers for Disease Control and Prevention. Manual does not indicate that the test was modified from its intended use.

3) In line 158 and elsewhere, the authors note that their prevalence data reflect a 50% decrease over those reported in the 2001-2004 cycle. This Reviewer finds the comparison interesting, particularly in the context of the current data being generated from a molecular assay (one that has received indications for testing of both symptomatic and asymptomatic individuals). This finding (i.e., decrease) warrants additional discussion; and, We appreciate this comment, and have added additional context in the discussion portion of the manuscript. The lower prevalence in this sample may be due to sample size, or perhaps new/different testing methods used in analysis of this sample in comparison to years past. Higher prevalence among females was consistent in our calculations in comparison to existing literature and also consistent in previous years of NHANES datasets.
4) In lines 226-228, the authors provide an initial discussion of the association between T. vaginalis and smoking status. More discussion is warranted, supported by peer-reviewed literature references.

Additional explanation of findings are discussed in lines 239-240, as well as three peer-reviewed articles on TV and other STIs are cited. We were also interested to find this correlation, and are excited to see that our calculated numbers from this dataset agree with the trend of smoking and TV infection found in other published works.
